# Retrospective clinical and genetic analysis of COL6-RD patients with a long-term follow-up at a single French center

**DOI:** 10.3389/fgene.2023.1242277

**Published:** 2023-12-13

**Authors:** Victor Morel, Frédérique Audic, Charlotte Tardy, Annie Verschueren, Shahram Attarian, Karine Nguyen, Emmanuelle Salort-Campana, Martin Krahn, Brigitte Chabrol, Svetlana Gorokhova

**Affiliations:** ^1^ Département de Génétique Médicale, Hôpital de la Timone, Marseille, Provence-Alpes-Côte d’Azur, France; ^2^ Service de Neuropédiatrie, Centre de Référence des Maladies Neuromusculaires de l’enfant PACARARE, CHU Timone, APHM, Marseille, France; ^3^ Inserm, U1251-MMG, Marseille Medical Genetics, Aix Marseille University, Marseille, France; ^4^ Centre de Référence des Maladies Neuromusculaires et de la SLA, ERN-NMD, CHU Timone, APHM, Marseille, France

**Keywords:** collagen type VI-related myopathies, COL6-RD, Bethlem myopathy, ullrich’s congenital muscular dystrophy, COL6 genes, *COL6A1*, *COL6A2*, *COL6A3*

## Abstract

Collagen type VI-related dystrophies (COL6-RD) are rare diseases with a wide phenotypic spectrum ranging from severe Ullrich’s congenital muscular dystrophy Ullrich congenital muscular dystrophy to much milder Bethlem myopathy Both dominant and recessive forms of COL6-RD are caused by pathogenic variants in three collagen VI genes (*COL6A1*, *COL6A2* and *COL6A3*). The prognosis of these diseases is variable and difficult to predict during early disease stages, especially since the genotype-phenotype correlation is not always clear. For this reason, studies with long-term follow-up of patients with genetically confirmed COL6-RD are still needed. In this study, we present phenotypic and genetic data from 25 patients (22 families) diagnosed with COL6-RD and followed at a single French center, in both adult and pediatric neurology departments. We describe three novel pathogenic variants and identify *COL6A2*:c.1970-9G>A as the most frequent variant in our series (29%). We also observe an accelerated progression of the disease in a subgroup of patients. This large series of rare disease patients provides essential information on phenotypic variability of COL6-RD patients as well as on frequency of pathogenic *COL6A* gene variants in Southern France, thus contributing to the phenotypic and genetic description of Collagen type VI-related dystrophies.

## Introduction

Collagen VI-related Dystrophies (COL6-RDs) are caused by pathogenic variants in the *COL6A1*, *COL6A2* and *COL6A3* genes and can be inherited in an autosomal dominant or recessive manner (Foley et al., 2021). These genes encode the three alpha chains of collagen type VI (α1, α2 and α3). Type VI collagen is a major component of the extracellular matrix, essential for the stability, nutrition, and development of muscle and connective tissue (Bönnemann, 2011). COL6-RDs represent a continuum of muscular disorders with large clinical spectrum consisting of three groups of COL6-RDs (Foley et al., 2021; Natera-de Benito et al., 2021). Ullrich congenital muscular dystrophy (UCMD) corresponds to the most severe phenotype, characterized by congenital weakness, hypotonia, proximal joint contractures, distal joints laxity and kyphoscoliosis. Age of onset is neonatal or early infancy. Some affected children acquire the ability to walk independently; however, progression of the disease results in a loss of ambulation in first decade, accompanied by generalized muscle atrophy and early respiratory failure (Foley et al., 2021). Bethlem myopathy (BM) is at the other end of the phenotypic spectrum. This much milder form of COL6-RD is characterized by a combination of proximal muscle weakness and joint contractures. There is mild to no developmental motor delay and all patients achieve ambulation. Neonatal features might be noted, but age of onset is usually childhood to early adulthood with evidence of slowly progressing weakness as well as contractures of the elbows, Achilles tendons, and long finger flexors (Foley et al., 2021). The intermediate form of COL6-RD often has a congenital onset, but the prognosis for the motor and respiratory impairment is better for these patients is better comparing to that of UCMD patients (Natera-de Benito et al., 2021). Certain clinical features are common to all COL6-RD subtypes, such as signs of distal laxity, skin damage (keloid scars, follicular hyperkeratosis and normal to mildly elevated levels of serum creatine kinase (CK) (Zanoteli et al., 2020; Foley et al., 2021; Natera-de Benito et al., 2021). Specific signs on muscle imaging can also be noted (Mercuri et al., 2005).

Interpretation of variants identified by genetic testing in COL6-RD patients can be quite challenging due to variable clinical presentation as well as presence of both recessive and dominant modes of inheritance for these disorders. More studies with phenotypic and genotypic description of COL6-RD patients are therefore needed. We report a new series of 25 French patients (22 families) with genetically confirmed COL6-RD. In this retrospective study, we describe the phenotypic and genotypic characteristics of COL6-RD patients with a long-term follow-up at the University Hospital of La Timone, Marseille. We define the frequencies of pathogenic *COL6A1-3* variants in the South of France and describe a subgroup of patients with an accelerated disease progression, thus contributing to better understanding of Collagen VI-related Dystrophies.

## Materials and methods

The patients were referred and followed at the University Hospital of Marseille (AP-HM, Marseille, France), in the Department of Adult Neuromuscular Disease and the Department of Pediatric Neurology. Patients F10, F14 and F15.1 were diagnosed by targeted Sanger sequencing of *COL6A1* and *COL6A2* genes after the diagnosis of COL6-RD was suspected due to presence of congenital hip dislocation, distal hyperlaxity or skin signs. Patients F5.2 and F15.2 were diagnosed by targeted Sanger sequencing since a pathogenic variant has been previously identified in the affected proband. The variants in the remaining 22 patients were identified by targeted exome sequencing (gene panel) using the genes on the National French Consensus Gene Lists for the diagnosis of myopathies (Krahn et al., 2019), as detailed in the Supplementary Table S1. Variants identified by high-throughput sequencing were first filtered using general population frequency, retaining only variants with <1% minor allele frequency in gnomAD v2.1.1 database. The remaining variants were then classified according to the ACMG/AMP guidelines as described in the Supplementary Material. As more information about pathogenicity of *COL6A1*, *COL6A2*, *COL6A3* variants became available over the years, initial classifications of certain variants have been modified.

All patients received standard care according to French National Protocol of Diagnostic and Care for COL6-RD established by the French Neuromuscular Disease Network (FILNEMUS, https://has-sante.fr/upload/docs/application/pdf/2022-10/pnds_col6_.pdf).

The study was conducted according to the guidelines of the Declaration of Helsinki. It was registered by AP-HM (Assistance Publique, Hôpitaux de Marseille) under number GBP3T5/PADS22-157, evaluating this study as exempt from the Institutional Review Board according to the French legislation. The appropriate consent was collected from each patient as part of standard procedure during diagnostic genetic testing in our laboratory. Additional written informed consent was collected for patients if detailed phenotype description was included in the manuscript.

## Results and discussion

We report 25 patients (22 families) with genetically confirmed COL6-RD. All of them have been followed at the University Hospital of Marseille (AP-HM), in the Department of Adult Neuromuscular Disease and the Department of Pediatric Neurology. Certain patients were initially followed in their childhood by the pediatric team and then transitioned to the adult care unit. The genetic diagnoses were established by the Medical Genetics Department of the same hospital for all patients except for F18. The close interactions between these three Departments as well as the unified medical record system allowed an efficient life-long follow up and care of the described COL6-RD patients, accompanied by a genetic diagnosis that in many cases became possible due to the arrival and evolution of high throughput sequencing technologies.

### Summary of genetic findings in our patient series

Fourteen patients (12 probands) were diagnosed with a recessive form of COL6-RD with bi-allelic variants confirmed by molecular testing (12 patients with homozygous variants and two patients with compound heterozygous variants). Eight different variants were responsible for these cases (two nonsense/frameshift, four splice-affecting variants and two missense variants). Eleven patients (10 probands) had a dominant COL6-RD, explained by a pathogenic or likely pathogenic heterozygous variant in one of the three *COL6A* genes. Eight different variants were identified in this group - five missense variants affecting Glycine residues in Gly-X-Y motifs of the triple-helical region of collagen and three in-frame deletions of one exon at the mRNA level (one large deletion and two splice-affecting single-nucleotide variants leading to exon skipping). Three of the identified pathogenic variants have never been published—one variant in the *COL6A1* gene (in-frame deletion of exon 3) and two variants in the *COL6A2* gene (c.1752del, p.(Gly585Glufs*11) and c.114_115 + 12del, p.(?)). The summary of all pathogenic and likely pathogenic variants identified in each patient is listed in Table 1, while detailed description of genetic evidence and ACMG/AMP codes used for variant classification is provided in the Supplementary Table S2.

**TABLE 1:** Variants identified in patients with COL6-RDs.

Patient	Phenotype class	Genomic coordinates (GRCh37)	Gene	Exon intron	Variant(s)	Variant type (consequence)	Alleles
Patients with bi-allelic variants (recessive)							
**F1**	B	chr21:47406990	*COL6A1*	In 5	c.717 + 4A>G; p.(?)	splicing	hom
**F2.1**	B	chr21:47409010	*COL6A1*	Ex 9	c.817A>T; p.(Lys273*)	nonsense	hom
**F2.2**	B	chr21:47409010	*COL6A1*	Ex 9	c.817A>T; p.(Lys273*)	nonsense	hom
**F3**	U	chr21:47531504_47531517	*COL6A2*	Ex 2	c.114_115 + 12del; p.(?)	splicing	hom
**F4**	B	chr21:47545690	*COL6A2*	In 25	c.1970-9G>A; p.(Thr656fs)	splicing	hom
**F5.1**	B	chr21:47545690	*COL6A2*	In 25	c.1970-9G>A; p.(Thr656fs)	splicing	hom
**F5.2**	B	chr21:47545690	*COL6A2*	In 25	c.1970-9G>A; p.(Thr656fs)	splicing	hom
**F6**	B	chr21:47545690	*COL6A2*	In 25	c.1970-9G>A; p.(Thr656fs)	splicing	hom
**F7**	U	chr21:47545690	*COL6A2*	In 25	c.1970-9G>A; p.(Thr656fs)	splicing	hom
**F8**	B	chr21:47545690	*COL6A2*	In 25	c.1970-9G>A; p.(Thr656fs)	splicing	hom
**F9**	B	chr21:47545690	*COL6A2*	In 25	c.1970-9G>A; p.(Thr656fs)	splicing	hom
**F10**	B	chr21:47545690	*COL6A2*	In 25	c.1970-9G>A; p.(Thr656fs)	splicing	comp het
		chr21:47544816		Ex 23	c.1752del; p.(Gly585Glufs*)	frameshift	
**F11**	I	chr21:47552300	*COL6A2*	Ex 28	c.2894G>C; p.(Arg965Pro)	missense	hom
**F12**	B	chr2:238253214	*COL6A3*	Ex 36	c.7447A>G; p.(Lys2483Glu)	missense	comp het
		chr2:238234368		Ex 43	c.9329-1G>T; p.(?)	splicing	
Patients with mono-allelic variants (dominant)							
**F13**	U	chr21:(47402678_47404182)_(47404384_47406439)	*COL6A1*	Ex 3	c.(227 + 1_228–1)_(428 + 1_429–1)del; p.(?)	in-frame exon deletion	het
**F14**	B	chr21:47407552	*COL6A1*	Ex 8	c.788G>A; p.(Gly263Asp)	missense	het
**F15.1**	U	chr21:47408998	*COL6A1*	Ex 9	c.805G>A; p.(Gly269Arg)	missense	het
**F15.2**	B	chr21:47408998	*COL6A1*	Ex 9	c.805G>A; p.(Gly269Arg)	missense	het
**F16**	B	chr21:47409016	*COL6A1*	Ex 9	c.823G>A; p.(Gly275Arg)	missense	het
**F17**	U	chr21:47409043	*COL6A1*	Ex 9	c.850G>A; p.(Gly284Arg)	missense	het
**F18**	I	chr21:47409881	*COL6A1*	In 11	c.930 + 189C>T; p.(?)	in-frame pseudoexon insertion	het
**F19**	I	chr21:47410741	*COL6A1*	In 14	c.1056 + 1G>A; p.(?)	in-frame exon skipping	het
**F20**	I	chr21:47410741	*COL6A1*	In 14	c.1056 + 1G>A; p.(?)	in-frame exon skipping	het
**F21**	I	chr21:47410741	*COL6A1*	In 14	c.1056 + 1G>A; p.(?)	in-frame exon skipping	het
**F22**	I	chr21:47535960	*COL6A2*	Ex 7	c.893G>A; p.(Gly298Glu)	missense	het
Patients with highly suspected COL6-RD, carrying a VUS							
**F23**	U	chr21:47421932	*COL6A1*	Ex 31	c.2014G>A; p.(Glu672Lys)	missense	hom
**F24**	B	chr21:47417650	*COL6A1*	Ex 22	c.1498G>A; p.(Gly500Arg)	missense	het
		chr2:238253214	*COL6A3*	Ex 36	c.7447A>G; p.(Lys2483Glu)	missense	het

All variants in this table are pathogenic or likely pathogenic, except for variants COL6A1 NM_001848.3:c.2014G>A; p.(Glu672Lys) and COL6A1 NM_001848.3:c.1498G>A p.(Gly500Arg). See Supplementary Table S1 for the detailed evidence used for ACMG/AMP, classification of variants. F, family; In, Intron; Ex, Exon; Hom, homozygous; comp het, compound heterozygous; het, heterozygous; U, ullrich congenital muscular dystrophy; I, intermediate form; B, bethlem myopathy; Y, yes; N, no; VUS, variant of uncertain significance. The following transcripts were used for variant nomenclature: *COL6A1* NM_001848.3, *COL6A2* NM_001849.4, *COL6A3* NM_004369.4.

Bold values indicates that families are numbered from 1 to 24 (F1–F24). .1 and .2 are used to differentiate patients within the same family.

**FIGURE 1:**
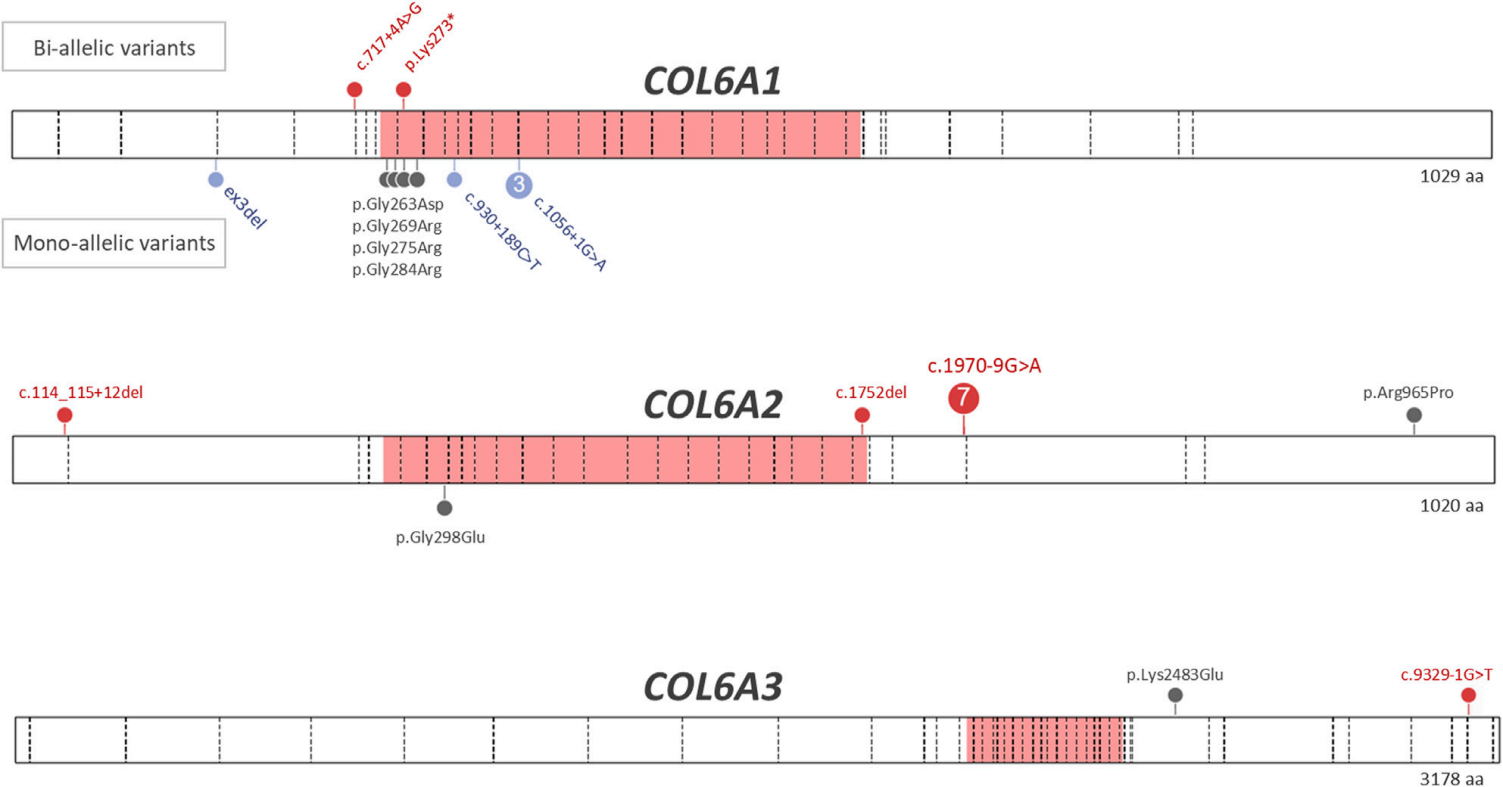
Variants identified in patients with COL6-RDs. Pathogenic and likely pathogenic variants identified in patients with genetically confirmed COL6-RD are shown. The triple helical domains in each collagen molecule are shown in pink. The exon-intron boundaries are marked by dashed lines. The missense variants are shown in grey, truncating variants are shown in red, while in-frame exon skips and deletions as well as in-frame pseudoexon insertions are shown in lavender. The bi-allelic (recessive) variants are shown above the protein bar, while the mono-allelic (dominant) variants are shown below the protein bar. The following transcripts were used for variant nomenclature: *COL6A1* NM_001848.3, *COL6A2* NM_001849.4, *COL6A3* NM_004369.4.

The numbers of patients with dominant and recessive COL6-RD were similar in our study, which is different from most previous COL6-RD cohorts where dominant cases were more prevalent (Briñas et al., 2010; Zanoteli et al., 2020; Natera-de Benito et al., 2021; Kwong et al., 2023). This difference is due to presence of seven probands carrying *COL6A2*:c.1970-9G>A variant on one or both alleles (six homozygous and one compound heterozygous). Similar to other COL6-RD cohorts, the patients with dominant COL6-RD in our series carried heterozygous glycine substitutions and in-frame events (exon skipping, exon deletion or pseudo-exon insertion). As in other studies, pathogenic glycine substitutions clustered in the N-terminal part of the triple helical domain (Figure 1). Glycine residue affecting 10th to 15th Gly-X-Y motifs of the triple helical domain tend to be more severe (Butterfield et al., 2013; Lamandé and Bateman, 2018). Moreover, heterozygous variants affecting the C-terminal part of the triple helical domain are found in the general population according to gnomAD database, suggesting that these variants are recessive or benign (Lamandé and Bateman, 2018). Collagen molecules with variants in the C-terminal part of the triple helical domain are thought to be excluded from the microfibril, while molecules with the variants in the N-terminal part are incorporated, thus exerting a dominant negative effect (Pace et al., 2008; Lamandé and Bateman, 2018). Two patients in our study carried variants in the N-terminal part of the triple region—*COL6A1*:c.850G>A p.(Gly284Arg) located in the 10th repeat (F17) and *COL6A2*:c.893G>A p.(Gly298Glu) located in the 15th repeat (F22). These two patients had Ullrich and Intermediate phenotypes respectively. Homozygous variants causing premature protein truncation in the triple helix domains are generally known to lead to most severe phenotypes (Briñas et al., 2010; Natera-de Benito et al., 2021). Interestingly, the only two patients in our series with a homozygous variant in this region (*COL6A1*:c.817A>T; p.(Lys273*)) were able to walk independently, even though they lost ambulation during childhood (patient F2.1, F2.2).

### Clinical findings in patients with genetically confirmed COL6-RD

The clinical features of 25 patients with genetically confirmed COL6-RD and two patients with highly suspected COL6-RD are summarized in Supplementary Table S1. Five genetically confirmed patients in our series were classified as Ullrich’s congenital muscular dystrophy (UCMD) (OMIM: 254090) by the clinical team, while fifteen patients had a milder Bethlem myopathy (BM) (OMIM: 158810). The remaining patients were affected by an Intermediate form of COL6-RD. For 10 patients, certain symptoms of COL6-RD, such as hypotonia, feet deformities or congenital hip dislocation, were evident at birth. Fourteen patients had their first symptoms appearing during infancy or childhood, while three patients had a much later adult onset of symptoms. Seven patients in our series lost the ability to walk, six of them during childhood (mean age = 8 years old). One patient had loss of ambulation at 57 years old. One patient has never acquired the ability to walk. In certain patients, a relatively mild initial disease course was followed by a phase of accelerated progression (marked as “+” in the “Accelerated Progression” column in the Supplementary Table S1). One of the patients that lost the ability to walk during childhood (at 10 years of age) and had an accelerated progression (F18) carried the COL6A1:c.930 + 189C>T deep intronic variant. Similar clinical presentation has been previously described for other carriers of this variant - these patients have a relative paucity of symptoms that then evolve rapidly to match a typical Ullrich Congenital Muscular Dystrophy with loss of ambulation around the age of nine, respiratory insufficiency and proximal contractures (Bolduc et al., 2019; Natera-de Benito et al., 2021). Another patient with an accelerated progression of symptoms and loss of ambulation during early childhood (4 years of age) carried the variant COL6A1:c.1056 + 1G>A (P21), while the other two patients with the same variant remained ambulant (F19 and F20). Interestingly, phenotypic variability has been previously described for this variant within the same family, with the mother presenting a relatively mild course of disease and the son showing an unusually fast progression with loss of ambulation during childhood (Lucioli et al., 2005). In comparison, all five patients carrying this variant in the recently published COL6-RD cohort had Bethlem myopathy (Natera-de Benito et al., 2021). Respiratory involvement was present in a third of patients (n = 8/24, data not available for one patient). Five of these patients needed bilevel positive airway pressure (BiPAP) (20.8% n = 5/24). Only two out of six UCMD patients had respiratory involvement. However, longer study is needed for the remaining four patients with normal respiratory function, as these patients are among the youngest of our series.

### Intrafamilial variability in our patient series

Our series includes three families—F2, F5 and F15. Two affected members of F2 are brothers carrying homozygous truncating variant COL6A1:c.817A>T; p.(Lys273*). The phenotypes of these patients were similar, as they both presented first signs in early childhood (with weakness of the lower limbs and frequent falls at 3 years for F2.1 and abnormal walking at 4 years for F2.2). They lost the ability to walk at 4 years of age for F2.1 and 9 years of age for F2.2. Both brothers underwent spinal arthrodesis at the age of 14. They also both needed respiratory support starting from 18 years of age for F2.1 and from 33 years of age for F2.2. F2.1 died at 40 years of age of acute respiratory failure. The variant found in these patients has been reported as homozygous in a patient with neonatal onset COL6-RD, mild CK elevation (350 U/L) and reduction of collagen VI in skin biopsy (Gonzalez-Quereda et al., 2020).

Two affected members of F5 are sisters carrying homozygous variant COL6A2:c.1970-9G>A; p.(Thr656fs). While both patients had the onsets during childhood (weakness of the lower limbs for F5.1 and loss of the ability to run at the age of 12 for F5.2), the phenotype was less severe for F5.2. The patient F5.2 was followed in our neuromuscular department since the age of 48, while her sister (F5.1) was followed from the age of 14. Neither of these two patients had respiratory or skin signs.

Two affected members of F15 are mother and son, both carrying a heterozygous missense variant COL6A1:c.805G>A; p.(Gly269Arg). They had a similar onset, with first signs of the disease during the acquisition of walking, diagnosed by frequent falls. The phenotype of the son (F15.1) is somewhat more severe, as he cannot get up from the ground without assistance. Both patients have retractions and similar skin signs such as keloid scars. F15.2 has required respiratory assistance since the age of 29, while F15.1 does not have respiratory involvement. The variant COL6A1:c.805G>A; p.(Gly269Arg) has been previously reported as appearing *de novo* in a patient with an intermediate form of COL6-RD with a neonatal onset (Deconinck et al., 2010). Possible explanations for the observed phenotypic variability include genetic context of the variant, with coding or non-coding variants in trans or in cis, as well as epigenetic or environmental contributions to the phenotype.

### Pathogenic variant frequencies observed in our patient series

Eight patients (seven unrelated families) in our series carried the variant *COL6A2*:c.1970-9G>A, either in a homozygous state (seven patients, F4-F9) or in a compound heterozygous state with a truncating variant c.1752del (one patient, F10), making it the most frequent variant in our series (29%). Indeed, the majority of patients with the recessive form of COL6-RD (7 out of 12 probands) carried the *COL6A2*:c.1970-9G>A variant. This variant introduces an aberrant splice acceptor site, leading to an insertion of seven nucleotides before exon 26 and resulting in a frame shift (Martoni et al., 2009; Foley et al., 2011). It has been identified in multiple patients affected with recessive COL6-RD, even though the frequency of this variant in published patient series is much lower than the one observed in our study (Quijano-Roy et al., 2014; Deconinck et al., 2015; Stehlíková et al., 2017). Similar to the previously described cases, the clinical presentation of patients with c.1970-9G>A was variable. For example, the age of onset ranged from birth to 35 years. Only four out of eight patients had retractions. The vast majority of these patients (7/8) were able to climb several steps without holding onto a railing. For now, none of the patients with c.1970-9G>A have lost the ability to walk.

It has been recently reported that a deep intronic variant c.930 + 189C>T inducing a pseudo-exon inclusion is a common pathogenic variant causing COL6-RD (Cummings et al., 2017; Bolduc et al., 2019). Indeed, this variant has been identified in up to 20% of patients with a suspected COL6-RD diagnosis and negative exon-based screening. Interestingly, this variant has been identified in only one patient in our series, even though the corresponding intronic region is now analyzed by our diagnostic gene panel. The frequency of the c.930 + 189C>T variant in COL6-RD patient series is thus variable depending on the population.

### Patient with variants in two different COL6 genes

One patient (F24) in our series carried two missense variants in two different *COL6A* genes - COL6A1:c.1498G>A, p.(Gly500Arg) and COL6A3:c.7447A>G, p.(Lys2483Glu). The first variant has never been described and is absent from the general population. Even though this variant affects a glycine residue in one of the Gly-X-Y motifs of the triple helical domain, this variant is located more C-terminally, outside of the mutational hotspot (Butterfield et al., 2013; Lamandé and Bateman, 2018). We therefore classified this variant as variant of uncertain significance (VUS, see Supplementary Table S2 for details). Indeed, several other glycine substitutions in the proximity of p.(Gly500Arg) are classified as VUS in ClinVar. The second variant, COL6A3:c.7447A>G, p.Lys2483Glu is a well-known pathogenic variant that has only been observed in patients with recessive COL6-RD (Villar-Quiles et al., 2021). Indeed, it is present *in trans* with a pathogenic variant in another patient in our series (F12). Neither of the two variants identified is likely to cause disease by itself. Interestingly, another affected relative of F24 also carries both of these variants, suggesting a possible case of digenic inheritance. Further exploration of this family would be necessary in order to determine the contribution of these variants to the patient’s phenotype. The digenic mode of inheritance has been previously described for a distal myopathy associated with a combination of one heterozygous variant in *TIA1* gene and one in *SQSTM1* gene (Niu et al., 2018). This mode of inheritance has also been suggested in a case report of a Bethlem myopathy patient carrying two putative *de novo* variants, one in *COL6A1* and one in *COL6A3* genes, though it is not clear if paternity and maternity were confirmed for the parental samples in that study (Choi et al., 2020). Exploration of the intronic regions is also necessary in order to rule out pathogenic variants that could be missed by the initial sequencing focused solely on exonic regions.

### COL6-RD-like patient carrying a homozygous variant of uncertain significance

In addition to the genetically confirmed COL6-RD cases, we describe a patient with a clinical presentation compatible with COL6-RD, carrying a homozygous variant of uncertain significance (F23). This patient presented with hypotonia, club feet and congenital hip dislocation at birth. He was able to sit at 8 months of age, but never acquired the ability to walk. He had severe muscle weakness predominantly involving proximal muscles, marked hyperlaxity in distal joints as well as scoliosis. His respiratory function and serum CK were normal. In addition to the muscular presentation, the patient also developed epilepsy at the age of 11 and psoriasis at the age of 14 that are currently treated and stable. Genetic testing identified a homozygous variant of uncertain significance in *COL6A1,* NM_001848.3:c.2014G>A; p.(Glu672Lys). This variant changes a highly conserved residue in the C1 domain and is predicted to have a deleterious effect on protein function (CADD = 25.8; REVEL = 0.762). It is present in the general population (POPMAX filtering allele frequency is 0.0003288 in the gnomAD v2.1.1 database, 16/30,506 alleles in the South Asian population without any homozygous individuals). ClinVar database has an entry for this variant, classified as variant of uncertain significance. Both parents of the patient are asymptomatic heterozygous carriers of this variant.

### Genetic tests used to diagnose COL6-RD patients

COL6-RDs represent a spectrum of muscular disorders from severe and more clinically recognizable Ullrich congenital muscular dystrophy (UCMD) to much milder Bethlem myopathy (BM) that can present as limb girdle muscular dystrophy (Foley et al., 2021; Natera-de Benito et al., 2021). It is for this reason that the *COL6A1-3* genes have been included on three different gene panels according to the French National Consensus Gene lists: “Limb Girdle Muscular Dystrophies (LGMD)” (Exhaustive gene list of 40 genes), “Congenital Muscular Dystrophies-except alpha-dystroglycanopathies” (Exhaustive gene list of 17 genes) and “Retractile Myopathies” (Unique exhaustive gene list of 29 genes) (Krahn et al., 2019). All four patients in our series that were screened by LGMD gene panel were diagnosed with Bethlem myopathy (Supplementary Table S1), while six Ulrich patients were diagnosed by “Retractile Myopathy” gene panel (patients F3, F13), “Congenital Muscular Dystrophies-except alpha-dystroglycanopathies” gene panel (F7, F17, F23) and by targeted Sanger sequencing (F15.1). These results show the importance of including *COL6A1-3* genes on gene panels that cover the full phenotypic spectrum of COL6-RDs. Analyzing *COL6A1-3* genes is especially important since COL6-RDs represent a significant part of genetic muscular disorders. In one large cohort, pathogenic variants in *COL6A1-3* genes were found to responsible for the phenotype of 97 out of 440 neuromuscular patients with confirmed genetic diagnosis (Sframeli et al., 2017). The diagnosis of COL6-RD was also established in 4% (55/1,259) and 8% (39/468) of patients with genetically confirmed LGMD (Nallamilli et al., 2018; Töpf et al., 2020).

## Conclusion

In this study we present phenotypic and genetic data from 25 patients (22 families) with genetically confirmed COL6-RD that are followed at the University Hospital of Marseille (AP-HM). This large series of rare-disease patients provides key information on the frequency of pathogenic variants in *COL6A* genes in Southern France. The phenotypic data from our study also show that a certain subgroup of COL6-RD patients can present with a rapidly accelerating clinical presentation. Our results thus contribute to the genetic and phenotypic description of COL6-RD that could be useful for design of future clinical trials.

## Data Availability

The datasets presented in this study can be found in online repositories. The names of the repository/repositories and accession number(s) can be found in the article/Supplementary Material.
