# Supplementary material for: Retrospective clinical and genetic analysis of COL6-RD patients with a long-term follow-up at a single French center
Source: Front Genet. 2023 Dec 13;14:1242277. doi: 10.3389/fgene.2023.1242277 (PMC10753780; doi:10.3389/fgene.2023.1242277)
Supplement: Supplementary file 1 [file Table1.pdf]

Supplemental Table S1. Clinical features of patients with COL6-RDs.

	Age/Sexe	Age of onset	First symptoms	Type	Years of follow up	Accelerated progression	Motor development	Rising from the floor unassisted	Ascending 4 steps	Ambulation status	Retractions	CDH, DH	Skin signs	Respiratory involvement	CK (UI/L)	CK	Additional Affected Family members	Genetic test
F1	12/F	Neonatal	CDH	B	7		Walking at 18m	+	+	Still walks	+	CDH, DH	KS	normal	672	3X	NA	MD
F2.1	40/M Deceased	Infancy (3y)	Legs weakness	B	34	+	normal	NA	-	Lost at 3y	+	NA, NA	NA	BIPAP (18y)	20	normal	3	MD
F2.2	41/M	Childhood (4y)	Gait impairment	B	31	+	normal	NA	-	Lost at 9y	+	NA, NA	NA	BIPAP (33y)	68	normal	3	MD
F3	3/M	At birth	Perinatal hypotonia, feet deformities, CDH	U	3		Walking at 3y	-	-	Still Walks	-	CDH, DH	-	normal	194	normal	NA	RM
F4	51/M	At birth	Feet deformity	B	2		normal	+	+	Still Walks (needs support 47y)	+	NA, NA	NA	normal	279	1.5X	NA	RM
F5.1	43/F	Childhood	Running difficulties	B	28		normal	+	+	Still Walks	+	N	-	normal	428	2X	2	LGMD
F5.2	51/F	Childhood	Running difficulties	B	3		normal	+	+	Still Walks	+	NA, NA	-	NA	421	2X	2	Sanger
F6	14/F	At birth	CDH	B	12		Walking at 2y 2 m	+	+	Still Walks	-	CDH, DH	-	normal	350	2X	NA	RM
F7	10/F	At birth	CDH, perinatal hypotonia	U	8		Walking at 4y 6 m	+	-	Still Walks	-	CDH, DH	KS, FK	normal	289	NA	NA	CM
F8	50/M	Adulthood (30y)	Legs weakness, difficulties climbing stairs	B	20		normal	+	+	Still Walks (needs support 40y)	-	NA, NA	NA	normal	305	1.5X	NA	LGMD
F9	54/M	Adulthood (35y)	Legs weakness, difficulties climbing stairs	B	19		normal	+	+	Still Walks (needs support 53y)	NA	NA, NA	NA	normal	650	3X	NA	LGMD
F10	46/M	At birth	CDH	B	38		normal	+	+	Still Walks	+	CDH	NA	normal	365	2X	-	Sanger
F11	9/M	Infancy (18mo)	Walk delay and gait impairment	I	6	+	Walking at 18 m	+	-	Still Walks	+	-	NA	BIPAP (9y)	720	4X	NA	CM
F12	65/M	Childhood	Difficulties running	B	9		normal	+	+	Still Walks	+	NA, NA	-	normal	600	3X	-	LGMD
F13	35/M	At birth	CDH	U	33		normal	NA	NA	Lost at 10y	+	CDH, NA	KS FK	BIPAP (32y)	916	5X	NA	RM
F14	61/M	Childhood (4y)	Legs weakness	B	46		normal	+	-	Lost at 57y	+	NA, NA	-	FVC : 41%	47	normal	4	Sanger
F15.1	9/M	Infancy (2y)	Falls	U	7		normal	-	-	Still Walks	+	DH	KS	FVC : 68%	239	1.5X	1	Sanger

F15.2	35/F	Infancy (2y)	Falls	B	28		normal	+	-	Still Walks	+	-	KS, FK	BIPAP (29y)	69	normal	1	Sanger
F16	63/F	Adulthood (51y)	Legs weakness	B	11		normal	+	+	Still Walks	-	NA, NA	NA	FVC : 63%	282	1.5X	2	MD
F17	9/M	At birth	Decreased Fetal Movements Perinatal hypotonia, Congenital Muscular Torticollis, CDH	U	9		normal	-	-	Still Walks	+	CDH, DH	KS	normal	363	2X	-	CM
F18	12/M	Infancy (13 mo)	Gait impairment, Falls	I	9	+	normal	+	-	Lost at 10y	+	DH	-	normal	278	1.5X	-	RM
F19	23/M	At birth	Feet deformity, CDH	I	11		normal	+	-	Still Walks	+	CDH, DH	KS,FK	normal	800	4X	-	MD
F20	15/M	Infancy (2y)	Falls	I	12	+	normal	+	-	Still Walks	+	CDH, DH	FK	normal	245	1.5X	-	CM
F21	5/M	Infancy (15mo)	Falls	I	5	+	normal	-	-	Lost at 4y	-	-	-	normal	302	1.5X	-	CM
F22	9/M	3 mo	CDH	I	8	+	Walking at 1y 6 m	+	-	Lost at 9y	+	CDH, DH	KS	normal	878	4X	-	RM
F23	16/M	At birth	Arthrogryposis Perinatal hypotonia, Feet deformity, CDH	U	16		Sitting at 8 m, several steps at 16y	-	-	Never acquired	-	CDH, DH	-	normal	188	normal	-	CM
F24	47/M	Childhood	Difficulties running	B	4		normal	+	+	Still Walks	+	NA, NA	-	normal	494	2X	1	MD

“Age” corresponds to the last clinical evaluation of the patient. For age of onset, the exact ages of onset are shown when available; “infancy” 0-3 years of age, “childhood” 4-18 years of age. Motor development was considered normal if sitting was achieved by 6 months of age and independent walking was achieved by 18 months of age.
“Accelerated progression” was considered present in a patient if the neurologist following the patient noted rapid aggravation of muscle weakness that was not expected given the previous course of disease in this patient.
“Ascending steps” refers to the capacity of patient to go up a minimum of 4 steps without use of a railing.
“Respiratory involvement” was noted for all patients with BiPAP had FVC < 40%
“Additional affected family members” reflects the total number of symptomatic individuals in addition to the proband, with or without genetic confirmation of COL6-RD
The column “Genetic test” if Sanger sequencing or targeted exome sequencing (gene panel) was used diagnosis, specifying the gene panel from the French National Consensus Gene lists (Krahn et al. 2019). LGMD – Limb Girdle Muscular Dystrophies - Exhaustive gene list (40 genes); CM – Congenital Muscular Dystrophies-except alpha-dystroglycanopathies - Exhaustive gene list (17 genes); RM - Retractile Myopathies - Unique exhaustive gene list (29 genes); DM – an earlier version of neuromuscular gene panel of 40 genes that was used for diagnosis before publication of National Consensus Gene lists.
F: Female; M: Male; y: years; mo: months; CDH: congenital dislocation of the hip; DH: distal hyperlaxity; U: Ullrich myopathy; I: intermediate form; B: Bethlem myopathy; NA: not available; +: Presence; -: Absence; Ks: keloid scars; fk: follicular hyperkeratosis; FVC: Forced Vital Capacity; CK: Creatine kinase (N: 20-200 UI/L), multiple of normal values is shown if CK is inscreased
