# Supplementary material for: Retrospective clinical and genetic analysis of COL6-RD patients with a long-term follow-up at a single French center
Source: Front Genet. 2023 Dec 13;14:1242277. doi: 10.3389/fgene.2023.1242277 (PMC10753780; doi:10.3389/fgene.2023.1242277)
Supplement: Supplementary file 2 [file Presentation1.pdf]

ACMG/AMP classification of variants

The following transcripts were used: *COL6A1* - NM_001848.3, *COL6A2* - NM_001849.4, *COL6A3* - NM_004369.4. The pathogenicity of variants was evaluated according to the ACMG/AMP criteria [1] with the following modifications. We applied the PVS1 criteria according to ClinGen Sequence Variant Interpretation (SVI) recommendations for Interpreting the Loss of Function PVS1 ACMG/AMP Variant Criteria [2], with the following modification: in-frame exon deletions or exon skipping events were attributed PVS1 for the following exons: COL6A1 – exons 8-14, COL6A2 – exons 5-12, COL6A3 – exons 15-18 [3]. The same regions were used to assign PS3_VS (very strong) if the exon skipping event was observed by RNA analysis. We applied PP3 score (*in silico* prediction of pathogenicity) if CADD or REVEL score were above 23 and 0.7 respectively. REVEL scores [4] were obtained from https://sites.google.com/site/revelgenomics/downloads and CADD scores [5] from https://cadd.gs.washington.edu/snv, using GRCh37-V1.6 model. SpliceAI score [6] of more than 0.5 was used to assign PP3 for non-canonical splicing variants. PM3 score (in *trans* with a pathogenic variant) was assigned according to the SVI Recommendation for in *trans* Criterion PM3 (Version 1.0, https://www.clinicalgenome.org/site/assets/files/3717/svi_proposal_for_pm3_criterion_-_version_1.pdf). For dominant cases, the following thresholds were used for PS4 assignment: PS4_sup if variant has been identified in 2 probands (proband reported here + another proband), PS4_mod - in 3-4 probands, PS4 – in 5 and more probands. PP1 score (segregation data) was assigned according to the recommendations by the Hearing Loss ClinGen Working group that focused in part on recessive disorders [7]. The thresholds for the allele frequency criteria were as follows: PM2 - 0.01%, BS1 - 0.1%, BA1 – 0.2%. The POPMAX Filtering Allele Frequencies were obtained from gnomAD v2.1.1 [8]. PP4 code was assigned if patient's phenotype or family history was highly specific for COL6-RD. The following elements were used to assign PP4 code: muscle weakness combined with distal laxity, skin manifestations (atrophic or keloid scars, follicular hyperkeratosis), or specific features in MRI such as concentric involvement of the vasti or 'central shadow' of the rectus femoris [9, 10]. PP4_moderate code was assigned for variants that were associated with abnormal collagen secretion and deposition in patient-derived fibroblast cultures. PP4 codes were only assigned to rare variants (PM2 also attributed). PM1 was attributed for missense variants changing glycine residues in the N-terminal part of the triple helical region: COL6A1 – residues 257-341, COL6A2 – residues 257-301, COL6A3 – residues 2039-2098 [3]. PP5 and BP6 were not used following the recommendations by ClinGen SVI Working Group [11]. All variants from this study were submitted to the Locus-Specific DataBases (LOVD 3.0) [12].

Supplemental References:

1 Richards S, Aziz N, Bale S, Bick D, Das S, Gastier-Foster J, Grody WW, Hegde M, Lyon E, Spector E, Voelkerding K, Rehm HL, ACMG Laboratory Quality Assurance Committee. Standards and guidelines for the interpretation of sequence variants: a joint consensus recommendation of the American College of Medical Genetics and Genomics and the Association for Molecular Pathology. *Genetics in Medicine: Official Journal of the American College of Medical Genetics* 2015;**17**:405–24.

2 Abou Tayoun AN, Pesaran T, DiStefano MT, Oza A, Rehm HL, Biesecker LG, Harrison SM, ClinGen Sequence Variant Interpretation Working Group (ClinGen SVI). Recommendations for interpreting the loss of function PVS1 ACMG/AMP variant criterion. *Human Mutation* 2018;**39**:1517–24.

3 Lamandé SR, Bateman JF. Collagen VI disorders: Insights on form and function in the extracellular matrix and beyond. *Matrix Biol* 2018;**71–72**:348–67.

4 Ioannidis NM, Rothstein JH, Pejaver V, Middha S, McDonnell SK, Baheti S, Musolf A, Li Q, Holzinger E, Karyadi D, Cannon-Albright LA, Teerlink CC, Stanford JL, Isaacs WB, Xu J, Cooney KA, Lange EM, Schleutker J, Carpten JD, Powell IJ, Cussenot O, Cancel-Tassin G, Giles GG, MacInnis RJ, Maier C, Hsieh C-L, Wiklund F, Catalona WJ, Foulkes WD, Mandal D, Eeles RA, Kote-Jarai Z, Bustamante CD, Schaid DJ, Hastie T, Ostrander EA, Bailey-Wilson JE, Radivojac P, Thibodeau SN, Whittemore AS, Sieh W. REVEL: An Ensemble Method for Predicting the Pathogenicity of Rare Missense Variants. *The American Journal of Human Genetics* 2016;**99**:877–85.

5 Rentzsch P, Witten D, Cooper GM, Shendure J, Kircher M. CADD: predicting the deleteriousness of variants throughout the human genome. *Nucleic Acids Res* 2019;**47**:D886–94.

6 Jaganathan K, Kyriazopoulou Panagiotopoulou S, McRae JF, Darbandi SF, Knowles D, Li YI, Kosmicki JA, Arbelaez J, Cui W, Schwartz GB, Chow ED, Kanterakis E, Gao H, Kia A, Batzoglou S, Sanders SJ, Farh KK-H. Predicting Splicing from Primary Sequence with Deep Learning. *Cell* 2019;**176**:535-548.e24.

7 Oza AM, DiStefano MT, Hemphill SE, Cushman BJ, Grant AR, Siegert RK, Shen J, Chapin A, Boczek NJ, Schimmenti LA, Murry JB, Hasadsri L, Nara K, Kenna M, Booth KT, Azaiez H, Griffith A, Avraham KB, Kremer H, Rehm HL, Amr SS, Abou Tayoun AN, ClinGen Hearing Loss Clinical Domain Working Group. Expert specification of the ACMG/AMP variant interpretation guidelines for genetic hearing loss. *Hum Mutat* 2018;**39**:1593–613.

8 Whiffin N, Minikel E, Walsh R, O'Donnell-Luria AH, Karczewski K, Ing AY, Barton PJR, Funke B, Cook SA, MacArthur D, Ware JS. Using high-resolution variant frequencies to empower clinical genome interpretation. *Genetics in Medicine: Official Journal of the American College of Medical Genetics* 2017;**19**:1151–8.

9 Foley AR, Mohassel P, Donkervoort S, Bolduc V, Bönnemann CG. *Collagen VI-Related Dystrophies*. University of Washington, Seattle 2021. https://www.ncbi.nlm.nih.gov/books/NBK1503/ (accessed 10 Apr2022).

10 Mercuri E, Lampe A, Allsop J, Knight R, Pane M, Kinali M, Bonnemann C, Flanigan K, Lapini I, Bushby K, Pepe G, Muntoni F. Muscle MRI in Ullrich congenital muscular dystrophy and Bethlem myopathy. *Neuromuscular Disorders* 2005;**15**:303–10.

11 Biesecker LG, Harrison SM, ClinGen Sequence Variant Interpretation Working Group. The ACMG/AMP reputable source criteria for the interpretation of sequence variants. *Genetics in Medicine: Official Journal of the American College of Medical Genetics* 2018;**20**:1687–8.

12 Fokkema IFAC, Kroon M, López Hernández JA, Asscheman D, Lugtenburg I, Hoogenboom J, den Dunnen JT. The LOVD3 platform: efficient genome-wide sharing of genetic variants. *Eur J Hum Genet* 2021;**29**:1796–803.
