# Supplementary material for: Retrospective clinical and genetic analysis of COL6-RD patients with a long-term follow-up at a single French center
Source: Front Genet. 2023 Dec 13;14:1242277. doi: 10.3389/fgene.2023.1242277 (PMC10753780; doi:10.3389/fgene.2023.1242277)
Supplement: Supplementary file 3 [file Table2.pdf]

Supplemental Table S2: The detailed evidence used for ACMG/AMP classification of *COL6A1-2-3* variants identified in our patient series

Variant	Evidence for each variant	ACMG/AMP Classification
COL6A1<br>NM_001848.3:c.(227+1_2281)_(428+1_429-1)del<br>p.(?)<br>exon 3<br>(GRCh37) chr21:<br>(47402678_47404182)_(47404384_47406439)	This variant has never been reported in the literature nor entered in the variant databases. It is an in-frame exon deletion that leads to deletion of 66 amino acids in a region close to the triple helix of *COL6A1*, where this type of deletions are well-known and are causative of autosomal dominant COL6A1-RD (PMID:20301676) [PVS1_strong]. Pathogenic variations of exon 3 3' splicing site inducing exon skipping have been described in patients with dominant form of COL6-RD (PMID:34167565 (2 probands, 1 family), PMID:25535305 (1 patient), PMID:11932968 (8 probands, 1 family), PMID: 15955946 (6 probands, 3 families), PMID: 28831785 (1 proband, 1 family). This variant is absent in the general population according to gnomAD v.2.1.1 [PM2_sup]. Our proband presents clinical characteristics of COL6-RD [PP4].	Likely Pathogenic (Class 4): PVS1_strong,<br>PM2_sup,<br>PP4
COL6A1 NM_001848.3:c.717+4A>G<br>p.(?)<br>intron 5<br>(GRCh37) chr21:47406990<br>Canonical Allele Identifier: CA10069654	This variant has been observed in a compound heterozygous state with a pathogenic variant in three patients affected with COL6-RD or presenting with limb-girdle weakness (LOVD individuals 00108711 (likely corresponding to one of the three patients in PMID: 32528171), 00109326 and PMID: 30564623) This variant was seen in a homozygous state in three patients (patient F1 from this study, LOVD #00109327 and PMID: 30564623). It was also found to be associated with a VUS in two patients (PMID: 30564623, PMID: 32065942). In eight additional individuals with this variant, the inheritance status or variant association data were not available (PMID: 32403337, PMID: 32528171, 5 ClinVar entries #284826) [PM3_strong]. This variant is rare in the general population according to gnomAD v2.1.1 (POPMAX filtering allele frequency: 0.0005%, 2/66712 alleles in European (non-Finnish) population) [PM2_sup]. This variant is predicted to affect splicing (SpliceAI : DL 0.55). The RT-PCR results are reported for one individual (LOVD #00109327) showing intron 5 retention, leading to a premature stop codon p.(Ile239fs*30) with NMD predicted [downgraded to PS3_mod since the actual data is not shown]. The probands present specific clinical characteristics of COL6-related myopathy [PP4].	Likely Pathogenic (Class 4): PS3_mod,<br>PM3_strong,<br>PM2_sup, PP4

COL6A1 NM_001848.3:c.788G>A: p.(Gly263Asp)<br>exon 8<br>(GRCh37) chr21:47407552<br>Canonical Allele Identifier: CA10605416	This variant has been reported once in a family with intra- and inter-generational phenotypic heterogeneity - mother with intermediate-COL6-RD, daughter mild COL6-RD, and very severe congenital onset in the son (Mendez del Barrio et al. 2016) [PP1]. This variant has been found in a heterozygous state and classified as likely pathogenic in two additional patients - one with LGMD presentation (PMID: 30564623, LOVD #00220669, ClinVar SCV000339604.4) and one with COL6-RD (LOVD #00108903). There are two additional entries for this variation in ClinVar (classified as likely pathogenic and VUS, Accession: VCV000286252.4) [PS4_mod]. Variants affecting the same amino acid (p.(Gly263Cys), p.(Gly263Val)) have been previously reported as pathogenic (PMID: 24038877, ClinVar) [PM5]. The variant identified in our patient is absent in the general population according to gnomAD v.2.1.1 [PM2_sup]. This variant is predicted to affect protein function (CADD v.1.6: 23.9; REVEL: 0.955) [PP3]. This variant is a substitution of a glycine in the triple helical domain of collagen, corresponding to a mutational hot spot [PM1]. The probands present specific clinical characteristics of COL6-related myopathy [PP4].	Likely Pathogenic (Class 4): PM1, PS4_mod, PM5, PM2_sup, PP1, PP3, PP4
COL6A1 NM_001848.3:c.805G>A p.(Gly269Arg)<br>exon 9<br>(GRCh37) chr21:47408998<br>Canonical Allele Identifier: CA10604778	This variant has been previously reported as appearing *de novo* in a patient with COL6-RD (PMID: 20576434). It was also identified in a heterozygous state and classified as "likely pathogenic" in a patient affected with COL6-RD and in a compound heterozygous state in a patient with limb-girdle muscular dystrophy according to LOVD database (Individuasl #00108884 and #00219792) [PM6, PS4_mod]. Another variant located at the same nucleotide and leading to the same amino acid change has been previously reported as pathogenic (c.805G>C, p.(Gly269Arg), PMID: 24038877) [PS1]. In addition, a variant affecting the same residue p.(Gly269Glu) has been previously reported as pathogenic (PMID: 29792937; 34167565) [PM5]. This variant is absent in the general population according to gnomAD v.2.1.1 [PM2_sup]. It is predicted to affect protein function (CADD v.1.6: 33; REVEL: 0.988) [PP3]. This variant is a substitution of a glycine in the triple helical domain of collagen, corresponding to a mutational hot spot [PM1]. This variant segregates with COL6-RD in one family (this study), present at heterozygous	Pathogenic (Class 5): PS1, PM1, PM5, PM6, PS4_mod, PP1, PM2_sup, PP3, PP4

	state in two affected individuals [PP1]. The proband presents specific clinical characteristics of COL6-related myopathy [PP4].	
COL6A1 NM_001848.3:c.817A>T p.(Lys273*)<br>exon 9<br>(GRCh37) chr21:47409010<br>Canonical Allele Identifier: CA410521439	This nonsense variant is located in the exon 9; NMD is predicted [PVS1]. In addition to one homozygous proband in our study, this variant has been reported as homozygous in a patient with neonatal onset COL6-RD and reduction of collagen VI in skin biopsy (PMID: 32403337) [PM3, PP4]. This variant segregates with COL6-RD in one family (this study), present at homozygous state in two affected individuals [PP1]. This variant is absent in the general population according to gnomAD v.2.1.1 [PM2_sup].	Pathogenic (Class 5): PVS1, PM3, PP1, PM2_sup, PP4
COL6A1 NM_001848.3:c.823G>A p.(Gly275Arg)<br>exon 9<br>(GRCh37) chr21:47409016<br>Canonical Allele Identifier: CA410521473	This variant has been previously reported in a family with three affected individuals (PMID: 15955946) [PS4_sup, PP1]. Another variant leading to the same amino acid substitution (c.823G>A p.(Gly275Arg)) is entered as “likely pathogenic” in ClinVar. Three other variants changing the same amino acid (c.824G>A p.(Gly275Glu), c.824G>T (p.Gly275Val) and c.823G>T p(Gly275Trp)) have been previously reported (PMID: 18378883, 32389683, LOVD, ClinVar) [PM5]. This variant is absent in the general population according to gnomAD v.2.1.1 [PM2_sup]. It is predicted to affect protein function (CADD v.1.6: 26.6; REVEL: 0.967) [PP3]. This variant is a substitution of a glycine in the triple helical domain of collagen, corresponding to a mutational hot spot [PM1].	Likely Pathogenic (Class 4): PM1, PM5, PP1, PM2_sup, PP3, PS4_sup
COL6A1 NM_001848.3:c.850G>A p.(Gly284Arg)<br>exon 9<br>(GRCh37) chr21:47409043<br>Canonical Allele Identifier: CA127115	This variant has been reported numerous times as pathogenic (PMID: 34008892 (1 proband), 24038877 (16 probands)) in patients with COL6-RD, either inherited from an affected parent or *de novo* (PMID: 24801232; 33060286, 24038877) [PS2_VS, PS4]. Immunostaining of muscle biopsies in individuals who were heterozygous for this sequence change revealed sarcolemma-specific collagen VI deficiency (PMID: 17785674)[PP4]. This variant is absent in the general population according to gnomAD v.2.1.1 [PM2_Sup]. This variant is predicted to affect protein function (CADD v.1.6: 29.6; REVEL: 0.975) [PP3]. This variant is a substitution of a glycine in the triple helical domain of collagen, corresponding to a mutational hot spot [PM1].	Pathogenic (Class 5): PS2_VS, PS4, PM1, PM2_sup, PP3, PP4
COL6A1 NM_001848.3:c.930+189C>T<br>r.930_931ins930+116_930+187	This variant is well known in the literature, reported in more than 30 patients, with *de novo* occurrences confirmed for 20 patients (PMID: 28424332, 30895940, 31607746) [PS2_VS, PS4]. Clinical phenotypes and muscle imaging	Pathogenic (Class 5): PS2_VS, PS3, PS4, PM1, PM2_sup, PP4

p.(Lys310_Gly311insTRSTAPRRPLHLEGQGQPPRHPAK)<br>intron 11<br>(GRCh37) chr21:47409881<br>Canonical Allele Identifier: CA658799468	were highly suggestive of COL6-RD (PMID 30895940) [PP4]. This variant is absent in the general population according to gnomAD v.2.1.1 [PM2_Sup]. Based on the RNA analysis, this intronic variant creates a cryptic splice donor site that leads to the inclusion of a pseudoexon of 24 amino acids within the well conserved N-terminal triple-helical region of COL6A1 [PS3, PM1].	
COL6A1 NM_001848.3:c.1056+1G>A<br>p.(?)<br>intron 14<br>(GRCh37) chr21:47410741<br>Canonical Allele Identifier: CA221748	This variant is well known in the literature (PMID: 10419498 (2 probands), 15689448 (4 probands, 3 families), 15955946 (12 probands, 6 families), 17886299 (2 probands), 27363342 (1 proband), 29419890 (3 probands), 25749816 (7 probands, 1 family), 24271325 (1 proband), 28831785 (2 probands, 1 family)) as well as in the ClinVar (Accession: VCV000017174.44) and LOVD databases. In total, this variant has been identified in the heterozygous state and classified as "likely pathogenic" or "pathogenic" in more than 30 patients with autosomal dominant COL6-RD [PS4]. This variant segregates with disease in an autosomal dominant fashion (PMID: 15955946, 25749816) [PP1_strong]. Two cases with this variant appearing *de novo* have also been described (PMID:28726809, 12840783) [PS2_VS]. This variant is present *de novo* in patients F19, 20 and 21. This variant is absent in the general population according to gnomAD v.2.1.1 [PM2_sup]. This variant is predicted to affect splicing (Splice AI: DL 0.90). Functional studies have shown that this variant causes in-frame skipping of exon 14, leading to a deletion of 18 amino acids in the triple helical domain (PMID: 10419498, 12840783) [PS3_VS]. Patients carrying the variant presented specific clinical characteristics of COL6-RD, with Collagen VI microfibrils markedly reduced in fibroblasts cultures from one patient (PMID: 12840783) [PP4_mod].	Pathogenic (Class 5):<br>PS2_VS, PP1_strong, PS3_VS, PS4, PP4_mod, PM2_sup
COL6A1 NM_001848.3:c.1498G>A p.(Gly500Arg)<br>exon 22<br>(GRCh37) chr21:47417650<br>Canonical Allele Identifier: CA410528805	This variant has never been reported in the scientific literature nor in the ClinVar and LOVD databases. It is absent in the general population according to gnomAD v.2.1.1 [PM2_sup].<br>This variant is predicted to affect protein function (CADD v.1.6: 26.7; REVEL: 0.943) [PP3]. Even though this variant affects a glycine residue in the triple helical domain, it is located more C-terminally, outside of the mutational hotspot (PMID: 24038877, PMID: 29277723, PM1 was not attributed). Indeed,	Variant of Uncertain Significance (Class 3):<br>PM2_sup, PP3

	several other glycine substitutions in the proximity of p.(Gly500Arg) are classified as VUS in ClinVar.	
COL6A1 NM_001848.3:c.2014G>A p.(Glu672Lys)<br>exon 31<br>(GRCh37) chr21:47421932<br>Canonical Allele Identifier: CA10070718	This variant has never been reported in the literature. ClinVar has one 'Likely Benign' entry for this variant (Variation ID: 1416827). The proband F23 is homozygous for this allele, while both parents of the patient are asymptomatic heterozygous carriers of this variant [PM3_sup]. This variant is relatively frequent in the general population according to the GnomAD v2.1.1 database (POPMAX filtering allele frequency is 0.03%, 16/30506 alleles in South Asian population, no homozygous). This variant is predicted to affect protein function (CADD v.1.6: 25.8; REVEL: 0.762) [PP3].	Variant of Uncertain Significance (Class 3): PP3, PM3_sup
COL6A2 NM_001849.4:c.114_115+12del<br>p.(?)<br>exon 2<br>(GRCh37) chr21:47531504_47531517	This variant has never been reported in the literature. It is absent from the variant databases as well as from the general population according to gnomAD v.2.1.1 [PM2_sup]. This 14-nucleotide deletion removes the 5' part of exon 2 thus deleting the canonical splice site donor site (SpliceAI : DL 0.99), likely leading to a frameshift with predicted NMD. Several other variants affecting the same splice site have been identified in multiple COL6-RD patients and reported as Pathogenic or Likely Pathogenic in ClinVar (#1324140, #476449) [PVS1]. This variant is present in a homozygous state in the proband F3 [PM3_sup]. The proband presents specific clinical characteristics of COL6-related myopathy [PP4].	Pathogenic (Class 5): PVS1, PM2_sup, PM3_sup, PP4
COL6A2 NM_001849.4:c.893G>A<br>p.(Gly298Glu)<br>exon 7<br>(GRCh37) chr21:47535960<br>Canonical Allele Identifier: CA410525356	This variant has been previously reported in a patient with an intermediate COL6-RD phenotype with highly specific features (PMID: 28760337), occurring *de novo* and present in a mosaic state, [PS2, PS4_sup]. Variants of the same residue have been reported as pathogenic: p.(Gly298Val) (PMID: 32528171, Accession: VCV001324137.1), p.(Gly298Arg) (ClinVAR Accession: VCV000210750.5), p.(Gly298Ala) (ClinVAR Accession: VCV000594119.4) [PM5]. This variant is absent in the general population according to gnomAD v.2.1.1 [PM2_sup]. It is predicted to affect protein function (CADD v.1.6: 29.9; REVEL: 0.984) [PP3]. This variant is a substitution of a glycine in the triple helical domain of collagen, corresponding to a mutational hot spot [PM1]. The proband presents specific clinical characteristics of COL6-related myopathy [PP4].	Pathogenic (Class 5): PS2, PM1, PM5, PM2_sup, PP3, PS4_sup, PP4

COL6A2 NM_001849.4:c.1752del p.(Gly585Glufs*11)<br>exon 23<br>(GRCh37) chr21:47544816<br>Canonical Allele Identifier: CA638187878	This variant has never been reported in the literature. It is very rare in the general population according to gnomAD v2.1.1 (no POPMAX filtering allele frequency is available, since only one allele is present (Ashkenazi Jewish population, 10028 alleles total, no homozygous)) [PM2_sup]. This variant is associated with the pathogenic variant c.1970-9G>A i*n trans* in the proband F10 [PM3]. It induces a frameshift causing a premature stop codon in the exon 23 that is part of a physiological transcript. NMD is predicted [PVS1]. The proband presents specific clinical characteristics of COL6-related myopathy [PP4].	Pathogenic (Class 5): PVS1, PM3, PM2_sup, PP4
COL6A2 NM_001849.4:c.1970-9G>A p.(Thr656fs)<br>intron 25<br>(GRCh37) chr21:47545690<br>Canonical Allele Identifier: CA10072396	This variant been previously described in homozygous or compound heterozygous state in more than 15 patients with phenotypes ranging from congenital Ullrich muscular dystrophy to Bethlem myopathy, through various intermediate phenotypes (PMID:19309692, 20576434, 27447704, 25535305, 24314752, 21280092, 29774307, ClinVar, LOVD). [PM3_strong]. This variant is rare in the general population according to gnomAD v2.1.1 (POPMAX filtering allele frequency: 0.022%, 15/35314 alleles in Latino/Admixed American population, no homozygous). This variant is predicted to affect splicing (SpliceAI AG: 0.85). This defect was confirmed by functional data. Transcript analysis showed a 7 nucleotide insertion between exons 25 and 26, resulting in a frame shift (PMID: 21280092) [PS3_VS]. Studies in patient fibroblast cultures demonstrated that the variant reduces levels of collagen VI protein, decreases intracellular secretion, and induces abnormal deposition and organization of the protein in the extracellular matrix (PMID: 19309692) [PP4_mod].	Pathogenic (Class 5): PS3_VS, PM3_strong, PP4_mod
COL6A2 NM_001849.4:c.2894G>C p.(Arg965Pro)<br>exon 28<br>(GRCh37) chr21:47552300<br>Canonical Allele Identifier: CA10588713	This variant has been previously reported in a homozygous state in four unrelated patients with limb-girdle muscular dystrophy presentation, classified as VUS (PMID: 30564623). It has also been identified in a patient *in trans* with a pathogenic variant c.1970-9G>A (DECIPHER Patient: 421133) as well as in another patient affected with Ullrich congenital muscular dystrophy, in trans with a likely pathogenic variant (ClinVar SCV002506997.1). Three additional entries are present in ClinVar for this variant (1 likely pathogenic, 2 VUS, VCV000265525.10) [PM3_strong]. This variant is rare in the general population according to gnomAD v2.1.1 (1/110998 alleles in	Likely Pathogenic (Class 4): PM3_strong, PM2_sup, PP3

	European (non-Finnish) population, no homozygous) [PM2_sup]. This variant is predicted to affect protein function (CADD v.1.6: 25.4; REVEL: 0.726) [PP3].	
COL6A3<br>NM_004369.4:c.7447A>G p.(Lys2483Glu)<br>exon 36<br>(GRCh37) chr2:238253214<br>Canonical Allele Identifier: CA244831	This variant has been identified in a compound heterozygous state with another pathogenic variant in more than 10 probands (PMID: 26247046 (1), 30706156 (1), 33749658 (10), 33596003 (1)). It has also been reported in a homozygous state in more than 10 probands (32448721 (2), 33749658 (4), 26247046 (1), 30706156 (3), 32403337 (2)) [PM3_Strong]. Clinvar has 11 entries for this variant with eight Pathogenic or Likely pathogenic classifications and three "variant of uncertain significance" classifications. This variant is relatively frequent in the general population according to gnomAD v2.1.1 (POPMAX filtering allele frequency: 0.09%, 133/129154 alleles in European (non-Finnish) population, 1 homozygous). It segregated with COL6-RD phenotype in several families where heterozygous carriers were unaffected (PMID: 33749658) [PP1_mod]. This variant is predicted to affect protein function (CADD v.1.6: 26.7; REVEL: 0.827) [PP3].	Likely Pathogenic (Class 4): PM3_strong, PP1_mod, PP3
COL6A3 NM_004369.4:c.9329-1G>T<br>p.(?)<br>intron 42<br>(GRCh37) chr2:238234368<br>Canonical Allele Identifier: CA351186089	This variant has never been described in another patient (the patient F12 from this study is the same as patient 7-II.1 in Villar-Quiles *et al*.2021, PMID:33749658). The variant is found in a compound heterozygous state with a likely pathogenic variant c.7447A>G (p.Lys2483Glu) [PM3]. This variant is absent in the general population according to gnomAD v.2.1.1 [PM2_sup]. It is located at the canonical splice site and is predicted to affect an acceptor splice site likely leading to an in-frame skipping of exon 43 (165bp, SpliceAI: AL 0.99) corresponding to a deletion of 55 amino acids [PVS1_strong]. The proband presents specific clinical characteristics of COL6-RD [PP4].	Likely Pathogenic (Class 4): PVS1_strong, PM3, PM2_sup, PP4
